# PINK1: A Bridge between Mitochondria and Parkinson’s Disease

**DOI:** 10.3390/life11050371

**Published:** 2021-04-21

**Authors:** Filipa Barroso Gonçalves, Vanessa Alexandra Morais

**Affiliations:** Faculdade de Medicina, Instituto de Medicina Molecular—João Lobo Antunes, Universidade de Lisboa, 1649-028 Lisbon, Portugal; filipagoncalves@medicina.ulisboa.pt

**Keywords:** PINK1, Parkinson’s disease, mitochondria homeostasis

## Abstract

Mitochondria are known as highly dynamic organelles essential for energy production. Intriguingly, in the recent years, mitochondria have revealed the ability to maintain cell homeostasis and ultimately regulate cell fate. This regulation is achieved by evoking mitochondrial quality control pathways that are capable of sensing the overall status of the cellular environment. In a first instance, actions to maintain a robust pool of mitochondria take place; however, if unsuccessful, measures that lead to overall cell death occur. One of the central key players of these mitochondrial quality control pathways is PINK1 (PTEN-induce putative kinase), a mitochondrial targeted kinase. PINK1 is known to interact with several substrates to regulate mitochondrial functions, and not only is responsible for triggering mitochondrial clearance via mitophagy, but also participates in maintenance of mitochondrial functions and homeostasis, under healthy conditions. Moreover, PINK1 has been associated with the familial form of Parkinson’s disease (PD). Growing evidence has strongly linked mitochondrial homeostasis to the central nervous system (CNS), a system that is replenished with high energy demanding long-lasting neuronal cells. Moreover, sporadic cases of PD have also revealed mitochondrial impairments. Thus, one could speculate that mitochondrial homeostasis is the common denominator in these two forms of the disease, and PINK1 may play a central role in maintaining mitochondrial homeostasis. In this review, we will discuss the role of PINK1 in the mitochondrial physiology and scrutinize its role in the cascade of PD pathology.

## 1. Introduction

Mitochondria are essential for life; they play an intimate role in almost all aspects of cellular function, since they are responsible for calcium intracellular homeostasis, metabolite biosynthesis, cell proliferation, differentiation and apoptosis, and, their most known purpose, energy production. This close interaction between mitochondria and the cell is the main reason why mitochondrial dysregulation is one of the underlying causes of several pathologies, such as cancer, neurological diseases and metabolic disorders. However, mitochondria are highly dynamic organelles that evoke several mechanisms to respond to bioenergetic challenges, such as biogenesis, events of fusion/fission and ultimately mitophagy, a pathway that controls their overall fate.

## 2. Mitochondria Homeostasis

Mitochondria are highly dynamic organelles that can change their morphological characteristics depending on their host cells environment and needs. Mitochondrial dynamics is a balance dictated by fission (division of one mitochondria by Drp1) and fusion (merging of different mitochondria by Mitofusins 1/2 and Opa1) events [1]. Interestingly, these events may occur to remove damaged portions within mitochondria or even to allow mitochondria movement to a high demanding metabolic area [1]. Additionally, changes in ATP/ADP ratio trigger mitochondria biogenesis, where PGC-1alpha is the major regulator of mitochondrial mass increase [2]. Important to note that all these processes require a coordination of several mechanisms, including expression of mtDNA genes, nuclear–mitochondrial communication and protein expression and import. Disruption of any of these processes can lead to a defective mitochondrial function. However, cells have evolved mechanisms to sequester and remove potentially damaged mitochondria, a form of autophagy called mitophagy [3]. As all these pathways are intertwined, one could argue that mitochondrial homeostasis and mitochondrial quality control are key regulatory features to maintain a healthy and robust pool of mitochondria in our cells. Consequently, when mitochondria go astray due to impairment of these pathways, the overall cell fate is dictated. A clear example of this is neurons, where mitochondrial dysfunction in these high energy demanding cells leads to neuron loss and ultimately to the development of neurodegenerative disorders.

At present, a complete picture of the molecular mechanisms involved in mitochondrial quality control is still to be unraveled. However, a key protein that has revealed to play a vital role in the regulation of several mitochondrial pathways is PINK1 (Phosphatase and Tensin homologue-induced kinase 1). This Serine/Threonine kinase is of special interest since it has different roles depending on the overall mitochondrial steady state, controlling this organelle’s fate [4]. Additionally, PINK1 is implicated in the etiology of Parkinson’s Disease (PD) as several mutations in this kinase are associated with early onset forms of this disease [5]. Thus, a detailed comprehension of PINK1’s biology will not only shed light on etiology of the neurodegenerative disease PD, but will furthermore lead to an enhanced understanding of the molecular pathways that regulate overall mitochondrial homeostasis.

Herein, we will look into the implications of PINK1 in mitochondrial physiology and scrutinize the role this protein plays in PD pathology.

## 3. PINK1 Helps Maintain Mitochondrial Homeostasis

*PINK1* gene was first identified in 2001, through a screen which aimed to analyze the PTEN unregulated targets in endometrial cancer cells [6]. This gene is localized at the human chromosome 1p36.12 and encodes for a Serine/Threonine kinase with 581 amino acid residues, and a molecular weight of approximately 63 kDa 4. *PINK1* was found to be regulated by Foxo3a, a downstream component of the PI3K/AKT pathway, which promotes *PINK1* transcription by binding to its promoter [7]. Additionally, by binding to its promoter, but under stress conditions, the nuclear factor κB up-regulates PINK1 transcription [8] More recently, it was described a downregulation of the *PINK1* gene by two different factors, p53 [9] and ATF3 [10].

**Figure 1:**
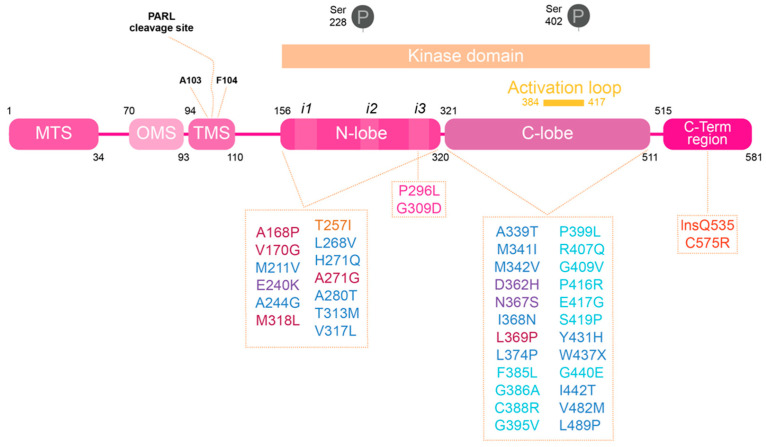
Human PINK1 domains and PD associated mutations. PINK1 is divided into different regions. At the N-terminal are the regions responsible for the processing and delivery of PINK1 to mitochondria: mitochondrial targeting sequence (MTS), the region recently called named the mitochondria membrane localization signal (OMS) and the transmembrane sequence (TMS). Within the TMS resides the PARL cleavage sites. The kinase domain is divided into an N-lobe and C-lobe; it is also the PINK1 domain where the majority of PD-associated mutations are found, and where there are the well described phosphorylation sites [15]. At the N-lobe there are the different inserts (i1, i2 and i3) identified in bioinformatics studies performed using PINK1 insects structure. The activation loop, at the C-lobe, changes the proteins conformation from inactive to active state upon phosphorylation. PINK1-PD mutations can be divided into mutations that affect PINK1’s structure, kinase activity or substrate binding, depending on residues and protein regions affected: ATP binding pocket (bordeaux), kinase core (dark blue), catalytic mutations (purple), insert 2 (yellow), insert 3 (pink), activation loop (light blue) and C-terminal region (red).

PINK1 is composed by a mitochondrial targeting sequence (MTS) located at N-terminal side (Figure 1). Additionally, in the N-terminal, we find what has been named the outer mitochondrial membrane localization signal (OMS), responsible for holding PINK1 to the outer mitochondrial membrane (OMM) upon depolarization [11,12]. The largest region of PINK1 is constituted by its kinase domain, region where the majority of identified mutations associated with PD have been described, a topic that we will elaborate later on this review. This kinase domain is constituted by N-lobe (residues 156–320 in human PINK1; hPINK1) and C-lobe (residues in 321–511 hPINK1). Finally, the C-terminal portion of PINK1 has been associated with the regulation of its kinase activity, since mutations on this terminal downregulate autophosphorylation, but seems to positively affect PINK1 kinase activity towards other substrates, such as histone H1 [13,14].

Despite being a widely studied protein, the resolution of human PINK1 crystal structure is a dilemma due to the poor solubility and rapid degradation of this protein [13,14,15]. To overcome this the field PINK1 insect orthologs (*Tribolium castaneum*, Tc; and *Pediculus humans corporis*) were used to perform structural studies, as they have high in vitro kinase activity [16] and share a kinase domain sequence identity of approx. 40% to the hPINK1 [17]. However, others have reported different substrate selectively between PINK1 orthologues [18]. Indeed, the experimental conditions used for TcPINK1 are not replicated when using a human form of PINK1, indicating probable regulatory differences between the two orthologues. While accessing these differences, Aerts et al. found that the full-length form of hPINK1 was not able to induce autophosphorylation, although it is catalytic active since it is able to phosphorylate PINK1 substrates, namely Parkin [19]. This suggests that the N-terminal region of PINK1 may have an inhibitory effect. The majority of studies demonstrating PINK1 kinase activity use truncated forms of PINK1, where parts of N-terminal are absent [13,14,15].

As reviewed by Rassol and Trempe, important mechanistic information on PINK1’s autophosphorylation and substrate binding were achieved, by the structural, biochemical and cellular studies done so far [20]. Bioinformatic and modelling approaches predicted that PINK1 carries additional regions when compared to other kinases, namely three inserts in the N-lobe and two flanking regions on its kinase domain: NT linker and CTE (C-terminal extension). This last one lies on the C-lobe, away from active site and has an important role in PINK1 stabilization [20]. This analysis also reveals that PINK1’s autophosphorylation is important to instigate a structural reorganization of insert 3 and consequently ubiquitin (Ub) binding, which needs two different articulations with PINK1, one with the N-lobe and other with the C-lobe, to form the active complex [20]. Although overall structure of PINK1 is likely conserved between species: there are relevant differences between the different animal models, for instance differences in inserts. The N-lobe of PINK1 has three inserts [15], and the length of insert 1 varies between species while insert 3 is the most conserved [16]. Intriguingly, PINK1 is not only a promiscuous kinase that selects its substrate based on the cellular environment it encounters, but it also seems like PINK1 has a different panoply of substrates depending on its host.

PINK1 is responsible for helping in maintaining homeostasis by interacting with several proteins. TRAP1 (TNF receptor associated protein 1/Heat shock protein 75) is an example, it plays a role in reducing reactive oxygen species (ROS) production and assisting protein folding [21,22]. Overexpression of PINK1 leads to an increase in phosphorylation of endogenous TRAP1 [23]. Other reports show an interaction with HtrA2 (high temperature requirement A2), also known as Omi, a protease released in the cytosol during apoptosis [24]; Htr2A is phosphorylated in a PINK1-dependent manner, since PINK1 siRNA leads to a decrease in Htr2A phosphorylation, although it is not clear if it is a direct phosphorylation [24].

Mitochondrial dynamics, a fundamental process to maintain a healthy mitochondrial pool, has shown to be regulated by PINK1. Studies performed using *Drosophila* showed that PINK1 downregulation lead to an increase in mitochondria length, resulting from excessive fusion, while PINK1 overexpression promoted mitochondrial fission [25]. The cytosolic dynamin GTPase Drp1, protein involved in fission events, is regulated by PINK1. It has been proposed that PINK1-mediated fission is dependent on the phosphorylation of Drp1 at residue S616, since in PINK1 null cells and mouse tissue a reduction in phosphorylation at this residue is observed [26]. Interestingly, when in presence of depolarized mitochondria, protein kinase A (PKA), which normally is recruited to OMM by AKAP1 to phosphorylate Drp1 at reside S637 and inhibit fission, is repressed by the presence and accumulation of PINK1, thereby ensuring the fission of damaged mitochondria [27]. Three putative PINK1-phosphorylation sites have been identified in Mitofusin 2 (Mfn2) at residues T111, S378 and S442. Interestingly, the phosphorylation of these residues leads to the suppression of mitochondrial fusion, even though residues T111 and S442 conjugated phosphorylation promotes Mfn2-Parkin binding [28]. Additionally, mitochondrial transport is regulated by PINK1, which can phosphorylate multiple sites of Miro, allowing normal mitochondrial movement in axon terminals [29,30].

The uptake of Ca^2+^ in mitochondria is highly important for several processes, such as oxidative phosphorylation or mitochondrial-induced apoptosis [31,32]. Several studies reveal a role of PINK1 in the regulation of mitochondrial calcium levels, where PINK1 deficiency leads to impaired Ca^2+^ levels in different models: cells [33], cultured neurons [34,35] and zebrafish [36] but affects Ca^2+^ homeostasis evoking different pathways. Heeman et al. reported that PINK1 depletion impaired Ca^2+^ uptake [33], whereas Gandhi et al. reported a regulation of Ca^2+^ levels by PINK1 via Na^+^/Ca^2+^ exchanger [34], where PINK1 deficiency causes an accumulation of Ca^2+^. More recently, dopaminergic neurons from a PINK1-null zebrafish model revealed an inhibition of mitochondrial Ca^2+^ uniporter (MCU) as being directly involved in the neurodegeneration process [36]. Still, new research has been encouraged to unravel this pathway.

Interestingly, PINK1 is enriched in Mitochondria Associated Membranes (MAMs) [37], a widely recognized site of Ca^2+^ exchange with the ER (endoplasmatic reticulum) [38], to maintain cellular bioenergetics and mitochondrial dynamics and transport [39]. Although PINK1’s involvement in mitochondria–ER tethering still remains to be explored, it was reported that upon CCCP treatment PINK1 accumulates on MAM’s and recruits Beclin1, a pro-autophagic protein, to generate omegasomes [37], which could suggest a new pathway for PINK1 in mitophagy. Additionally, fibroblast derived from patients with PINK1 mutations exhibit an increased ER–mitochondria co-localization, resulting in an abnormal Ca^2+^ signaling [40].

Without a doubt PINK1 is vital for overall mitochondria homeostasis as it regulates several mitochondrial-related pathways within the cell. However, what dictates which pathway will be ameliorated in PINK1 loss-of-function scenarios remains to be clarified.

## 4. PINK1 in Healthy Mitochondria

**Figure 2:**
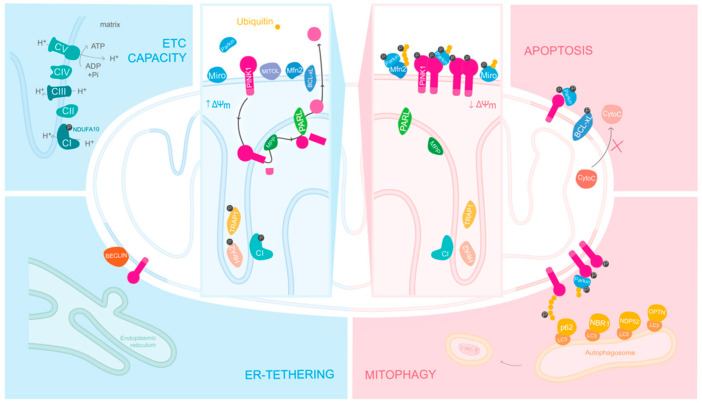
PINK1 has different roles depending on mitochondria’s overall state. In the presence of healthy mitochondria, PINK1 is internalized and phosphorylates, among other substrates, the complex I subunit NDUFA10 at the inner mitochondrial membrane (IMM). By regulating the enzymatic activity of complex I, PINK1 modulates the overall electron transport chain (ETC) capacity and, ultimately, the overall output levels of ATP. Afterwards, PINK1 is sequential cleaved by the proteases MPP and PARL and released to the cytosol for degradation. PINK1 also has a protective role, as it phosphorylates Bcl-xl in order to inhibit apoptosis. When mitochondria are depolarized, PINK1 accumulates on the outer mitochondrial membrane (OMM), where it forms homodimers and undergoes autophosphorylation. After this, PINK1 recruits and phosphorylates Parkin, and consequently also phosphorylates ubiquitin. Due to Parkin’s E3 ubiquitin ligase activity, Parkin and PINK1 create a positive feedback-loop, recruiting more ubiquitin and Parkin to be phosphorylated, creating poly-ubiquitin chains all around the surface of damaged mitochondria. This targets mitochondria for degradation via mitophagy. Posteriorly, due to the recruitment of autophagic receptors, like LC3, OPTN and NDP52, damaged mitochondria are engulfed and degraded via autophagy. Upon depolarization, ER-tethering to mitochondria is also hampered as PINK1 accumulates on MAM structures recruiting Beclin1 to form omegasomes, which are autophagosome precursors. ΔΨm, mitochondrial membrane potential; CI, Complex I; CII, Complex II; CIII, Complex III; CIV, Complex IV; CV, Complex V or ATP synthase; P, phosphorylation.

PINK1 has been described as a sensor of the overall health status of mitochondria, as the localization and substrate specificity of this kinase is defined when it encounters healthy versus unhealthy mitochondria (Figure 2).

In steady state, PINK1 is targeted to the mitochondria via its MTS. It has been established that MTS-carrying proteins interact with the translocase of outer and the inner membrane (TOM20 and TIM23, respectively) [41]. Along these lines, the conventional theory assumes that PINK1, being a MTS carrying protein, passes through these translocases and enters the matrix, where the MTS is cleaved by a mitochondrial processing protease (MPP) [42]. Then, a second cleavage occur by the mitochondrial rhomboid protease PARL (Presenilins-associated rhomboid like protease), between Ala103 and Phe104 in the transmembrane stretch, reducing the protein size to a 52kDa processed form [43,44,45], which is released in the cytosol and later degraded by the proteasome [46]. Consequently, PINK1 has a high turnover rate, and it is maintained at a low expression levels within the tissues [47,48].

Despite the increasing number of reports, there are still some conflicting data about the processing of PINK1 and localization of the kinase domain sub-mitochondrially. In fact, in the previously explained processing events, it is not clear how PARL, that resides in the intermembrane mitochondrial space (IMS), can cleave a protein that is associated with the TIM channel. Can PINK1 be partially imported? Some studies have shown that PINK1 or processed forms of PINK1 are located inside mitochondria [13,42,43,49]. While others have shown a partial PINK1 import, only for MPP and PARL cleavage to occur, and the kinase domain remains at the outer membrane facing the cytosol [46,50]. A few reports show that m-AAA proteases can be involved in this second cleavage of PINK1, helping its translocation in the inner mitochondria membrane (IMM) producing a form accessible to PARL [42]. On the other hand, Liu et al. show that PINK1, after being cleaved by PARL, suffers major conformational changes that allow the ubiquitination at Lys137, though an unknown E3 ubiquitin ligase [51]. More studies must be done to clarify PINK1 import and proteolytic processing inside mitochondria. By now we know that PINK1 regulates itself by autophosphorylation, since two phosphorylation sites have been reported, Ser228 and Ser402. Important to stress that Ser402 is located in PINK1’s activation loop, a crucial region to kinase activity even towards PINK1 substrates, contrarily to Ser228 that seems to be dispensable. There are other two putative phosphorylation sites, T313 and T257. Interestingly, the substitution of threonine in residue 313 to a methionine (T313M) is described as a PD mutation, which created a higher curiosity around this residue but, like T257, it does not affect PINK1 autophosphorylation.

Several studies have demonstrated altered mitochondria phenotypes in loss of function PINK1 cells and animal models [52,53,54,55]. Drosophila PINK1 mutant displays motor disturbances, abnormal synaptic transmission, structural mitochondrial changes and decreased membrane potential [52,53]. These different phenotypes were later explained as downstream consequences of a reduced Complex I enzymatic activity in Drosophila and mouse models [55]. Moreover, Morais et al. demonstrated with this study the relevance of PINK1 for overall electron transport chain (ETC) function, since wild-type (WT) hPINK1, but not a kinase inactive form of PINK1, was able to fully restore the enzymatic activity of Complex I in PINK1-null models. At present, all evidence points out that when PINK1 encounters a healthy mitochondria, it is internalized, processed and able to phosphorylation mitochondrial-resident substrates, such as the Complex I subunit NDUFA10.

## 5. PINK1 in Unhealthy Mitochondria

Across the IMS, essential for mitochondrial function, there is a membrane potential, which regulates the ETC and consequently the generation of ATP. In several cases of mitochondria damage, a loss of this potential occurs. When PINK1 encounters depolarized mitochondria its import is halted, and an accumulation of the full-length form occurs on the OMM [56,57]. In this case, PINK1 kinase activity suffers a shift, resulting in autophosphorylation on Ser228 and Ser402 and PINK1 dimerization [19,58,59]. Afterwards, one of the most studied PINK1 interactions occurs, an event first identified in Drosophila as a genetic link to an E3 ubiquitin ligase Parkin [52,53]. Parkin, largely known to cooperate with PINK1 in the removal of damaged mitochondria, interestingly is also known to be mutated in early onset PD cases [60,61]. The Drosophila studies reported that both PINK1 and Parkin mutant flies exhibit indirect flight muscle and dopaminergic neuronal degeneration accompanied by locomotive defects [52,53]. All these defects in PINK1 mutant flies were restored when Parkin was re-introduced. However, the reintroduction of PINK1 did not restore Parkin mutant fly defects, clearly indicating that Parkin works downstream of PINK1.

### 5.1. PINK1/Parkin Mediated Mitophagy

The crystal structure of Parkin revealed that it naturally exists in an autoinhibited conformation [62,63]. However, PINK1 phosphorylated ubiquitin (pUb) molecules interact with Parkin, altering its structure and making Ser65 of the ubiquitin-like domains (UBL) available for PINK1-mediated phosphorylation [64,65]. Only after these two steps is Parkin fully activated. In PINK1-null mammalian cells, Parkin recruitment is completely abolished, and is rescued when a WT but not a kinase-inactive form of PINK1 is re-introduced, confirming that Parkin needs an active PINK1 to be recruited to depolarized mitochondria [56,57,58,59,60,61,62,63,64,65,66].

Parkin’s ligase activity will recruit more ubiquitin and Parkin molecules to be phosphorylated by PINK1, creating poly-ubiquitin chains on the OMM [67]. This will further lead to an interaction with autophagy receptors containing an ubiquitin-binding domain and the LC3-interacting region, such as OPTN, NDP52 and NBR1 [68,69,70], that once connected to the autophagosome membrane will lead to mitochondrial clearance via mitophagy. The p62 or sequestome-1 (SQSTM1) role in this pathway is still controversial, while a number of studies demonstrate that it is recruited to depolarized mitochondria [71,72], others show that it is not essential for mitophagy to occur [73].

Supplementary to mitophagy, some mitochondrial proteins that are ubiquitinated by Parkin are degraded, such as Mitofusin and Miro1 leading to an impaired mitochondrial network [74,75] and mitochondrial movement [29,76,77], respectively.

Important to note that how PINK1 is stabilized on the OMM still remains to be clarified. Previous studies using BN-PAGE analyses revealed that PINK1 accumulates on the OMM in a phosphorylated form and is associated with the TOM40 complex [59,78]. More recently, Sekine et al. suggested that TOM7 may also play a role, as in TOM7 knockout (KO) cells both PINK1 and Parkin recruitment were defective after mitochondrial depolarization treatment [12].

Besides PINK1 stabilization, it is also not clear the mechanism by which Parkin is recruited to mitochondria. A recent study [79] proposes that MITOL (mitochondrial ubiquitin ligase: also known as MARCH5) can ubiquitinate proteins at the OMM, which will facilitate the recruitment of Parkin. Other studies suggest that the phosphorylation of proteins on mitochondria surface by PINK1 will work as “Parkin receptors”, like for instance ubiquitin [80], Mfn2 [81] or Miro [77].

Besides the putative phosphorylation of Htr2A in the “healthy mitochondria” pathway, PINK1 has also a protective role against apoptosis upon depolarization, when phosphorylating BCL-xL [82]. This anti-apoptotic protein is localized at OMM, where in a phosphorylated form it prevents the release of cytochrome c and caspases, inhibiting apoptosis [83].

Additionally, in these “unhealthy” mitochondria pathways there was described a link to PD. Zhu and collaborators observed mitochondria in autophagossomes in neurons of PD patients [84]. Later on, several groups reported abnormal mitophagy in different PD models [85,86,87], facts that we will further explore in the next topic of this review, suggesting a link between a PINK1 regulated pathway and PD.

### 5.2. PINK1 Independent Mitophagy

Important to note that in the past few years a couple of papers show that PINK1 may not be necessary for mitophagy. In 2017, Koentjoro discovered a mitochondrial autophagy receptor Nip1-like protein X (Nix) responsible for isolating mitochondria into autophagossomes in derived fibroblast from an asymptomatic patient carrying a homozygous Parkin mutation, where all mitochondrial functions and dynamics, including mitophagy, were working [88]. Moreover, Koentjoro showed that Nix compensates for the loss of PINK1/Parkin, as overexpression of Nix restores activation of mitophagy and mitochondrial energy production. Yet, Nix mediated mitophagy, as well as the expression of this protein in PD patient brains or animal models, remains to be determined. McWilliams’ team studied the function of PINK1 in vivo using a mito-QC reporter mice, which allows the visualization of mitophagy and mitochondrial architecture [89]. Analyzing PINK1 WT or KO mice crossed with mito-QC, they reported that PINK1 was present in all regions of the central nervous system, and that loss of PINK1 did not affect basal levels of mitophagy, when compared to WT mouse brains. Giving the hint that PINK1, mitophagy can occur in a PINK1 independent manner [89]. Nevertheless, it cannot be concluded that PINK1 is dispensable for all forms of mitophagy, indeed the precise role of this protein in vivo still remains to be defined, and PINK1’s function may be cell type and content dependent.

Despite this, these recent findings only confirmed the complexity of this process. Therefore, we believe that the study of physiological mitophagy and its alteration when triggered with pathological cascades needs further investigations.

## 6. PINK1 in PD Pathogenesis

Parkinson’s disease is the second most common neurodegenerative disorder, characterized by the loss of dopaminergic neurons (DA) in the substancia nigra and the formation of cytoplasmic inclusion bodies containing alpha-synuclein, called Lewy Bodies (LB). When about 70% of neurons are lost, the communication between brain and muscle cells weakens, resulting in the classic motor symptoms as the resting tremor, stiffness, bradykinesia and postural instability [90]. The etiology of this disease remains to be clarified but is considered to be the result of a combination of genetics and environmental factors, and where aging also has a crucial role. Indeed, PD cases are divided into sporadic and familiar; these last ones carry a heritable disease mutation in genes referred to as PARK genes, and account for 5–10% of all the PD cases [91].

### 6.1. PINK1 as a Genetic Cause of PD

Mutations in several of these PARK genes also cause mitochondrial dysfunctions, namely PINK1 (PARK6) 5 and Parkin (PARK2) [60,61]. PINK1 mutations cause autosomal recessive PD and certain clinical features are more common in patients with PINK1 mutations. For example, the outstanding difference is the age of onset that in this case is around 30 years of age. Additionally, patients harboring these mutations experience a more benign course with slower progression, more gait and balance difficulties, a better response to L-DOPA and increased dyskinesias [92,93]. Most patients with PINK1-associated PD experience a good response to levodopa and they do not typically develop dementia, although some studies report psychiatric features [94]. It is important to point out the fact that PINK1 was detected in some LBs in sporadic cases as well as in samples carrying only one allele mutant for PINK1, which are clinically and pathologically indistinguishable from the sporadic form [49].

To date it has been reported in the Human Gene Mutation Database (HGMB) at least 130 mutations in the *PINK1* gene, and they can be homozygous or heterozygous; missense mutations, truncating mutations and exon rearrangements [5,16]. A large majority of PINK1 mutations are in kinase domain, demonstrating the importance of PINK1 kinase activity in PD pathogenesis (Figure 1). The firstly identified PINK1 mutations were reported back in 2004 in Spanish (G309D) [5], Italian (W437X) [5], Filipino (L347P) [95], Japanese and Israeli families (R246X/H271Q) [95]. When in 2017 Schubert et al. revealed the structure of PhPINK1 in complex with ubiquitin [96], they were able to pinpoint the location of dozens of PINK1 PD causing mutations (Figure 1). The majority of them are in the kinase core and affect the protein fold or in the activation-loop. Mutations known to affect catalysis were mapped in the ATP binding pocket. These observations are in concordance with previous observations where, for instance, G309D mutations described as in insert 3 are largely described with defective kinase activity [16]. As we already described, PINK1 is involved in several pathways, and some have been proven to be altered in PD.

### 6.2. PINK1’s Link to Dysfunctions in the Respiratory Chain

The most known and described correlation between mitochondria and PD, is the injection of a synthetic heroin, 1-methyl-4-phenyl-1,2,5,6-tetrahydropyridine (MPTP) that rapidly generated Parkinsonism symptoms in drug abusers [97]. This compound has the ability to pass the blood–brain barrier, where it is oxidized to MPDP^+^ by monoamine oxidase B in glia and serotonergic neurons and then is converted to MPP^+^. This metabolite has a high affinity to dopamine transporters and causes an inhibition of Complex I, and preferentially mediates the degeneration of SN dopaminergic neurons [98,99]. The correlation between mitochondria impairment and PD was later validated when reduced activity in complex I, II and IV was found in brain, skeletal muscle and platelets of patients with sporadic PD [100,101]. Posteriorly, also some pesticides and herbicides, namely rotenone and paraquat that selectively inhibit Complex I, were also shown to cause Parkinsonism in animal models [102].

Several reports that analyzed fibroblasts from patients harboring PINK1 mutations revealed alterations in the ETC and overall oxidative stress levels [103,104,105]. PINK1 deficient mice reveal impaired dopamine release [106], compromised mitochondrial respiration, increased sensitivity to oxidative stress, progressive weight loss and selective reduction in locomotor activity in older animals [107]. Indeed, Morais et al. demonstrated that the key defect in ETC at the level of Complex I in PINK1 KO mice was due to reduced phosphorylation of the Complex I subunit NDUFA10 [106], confirming that PINK1 phosphorylation is required for Complex I function at the level of ubiquinone reduction. Phosphorylation at this site is a prerequisite for ubiquinone reduction, critical to mitochondrial bioenergetics and ROS production. ROS develops when electrons escape the ETC, especially at complexes I and III [107]. Interesting is the link between dopamine metabolism and ROS, where dopamine degradation causes an increase in ROS production [108], revealing a pathogenic cascade from mitochondrial impairment to neuronal dysfunction, which could explain neuronal death. Additionally, reduced enzymatic activity of Complex I has also been observed in several PD patients. Indeed, an impaired Complex I was one of the first observations made in sporadic PD patients [109], providing a link between mitochondrial dysfunctions and PD. However, whether NDUFA10 phosphorylation is reduced in PD patients harboring PINK1 mutations is still to be shown.

### 6.3. PINK1 in Mitophagy Context

Mitophagy is a key pathway for maintenance of mitochondrial health and, consequently, to maintain neuronal health. An impaired mitophagy results in an accumulation of damaged mitochondria, leading to neuronal death and consequent neurodegeneration, indeed an abnormal mitophagy has already been linked to neurodegeneration [110]. This pathway was confirmed in PD patient-derived fibroblasts and iPSC-derived neurons [111,112,113]. Moreover, Lewy bodies contain aggregates of alpha-synuclein, ubiquitin, and other compounds [114], including mitochondria. One study has reported mitochondria within autophagossomes in neurons of PD patients [84]. Abnormal mitophagy was posteriorly observed in several PD models [85,86,87], but the analysis of mitophagy has to be done with extreme caution, since Rakovic and collaborators demonstrate that the mitophagy process differs between human non-neuronal and neuronal cells, and that the comparison between models with endogenous Parkin expression versus exogenous expression may be misleading [112]. Moreover, the use of different experimental approaches to assess mitophagy have also contributed to contradictory findings in the field [115]. Studies performed in *Drosophila* using mt-Keima, a pH-dependent fluorescent protein fused to a mitochondrial targeting sequence, have shown that mitophagy occurs in muscle and dopaminergic neurons of aged flies [116]. However, in young adult flies no defects in mitophagy are observed, suggesting a role for PINK1 in age-dependent mitophagy. On the other hand, studies using mito-QC, a tandem mCherry-GFP tag fused to a mitochondrial targeting sequence, showed that basal mitophagy is readily detectable and abundant in many tissues of *PINK1* deficient flies [117]. These findings provide evidence that, at least in these experimental conditions, PINK1 and parkin are not essential for basal mitophagy in *Drosophila*, further underpinning that the molecular mechanisms of basal mitophagy remain largely obscure. Nevertheless, fibroblast from PINK1-PD patients confirmed data obtained with other PINK1 models, such as the stabilization and recruitment of Parkin on depolarized mitochondria [105]. Additionally, iPSC-derived neuron cells from an individual with PINK1-PD mutations reported an impaired recruitment of Parkin to mitochondria, and increased mitochondrial copy number [113], indicating that clearance of defective mitochondria is hampered. The relationship between mitochondria autophagy impairment and its contribution to pathogenesis of PD seems more and more evident, but regarding the PINK1/Parkin pathway, although it is critical to regulate mitophagy, the overall mechanism is still unclear. We believe that modulation of this pathway may have a therapeutic potential; nevertheless, investigation is still needed.

## 7. Conclusions

PINK1 has a pivotal role in several mitochondrial processes. One of these processes is the mitochondrial quality control pathway, known to be related to several diseases, as neurodegenerative diseases, cardiovascular diseases, diabetes or obesity. However, the exact mechanism behind most of PINK1 pathways still remains to be clarified, mostly because of PINK1 low expression levels and fast turn-over. This is something that the field has been working and debating for a while, and at present several working tools are being developed to further strengthen these findings.

Parkinson’s disease is the second most common neurodegenerative disorder, thus clarification of its pathogenic mechanism and the development of new diagnostic approaches and effective therapeutics are eagerly awaited. Almost 20 years after PINK1 was linked to this disease a lot of research has characterized this protein and its mutations associated to PD. The discovery of PINK1’s link to Complex I activity was one of the major breakthroughs in the field. Important to note is that Complex I was linked to sporadic cases of PD before the PINK1 connection to this disease, as a deficiency in Complex I has already been reported in the *substantia nigra pars compacta* (SNpc) of PD patients. More recently the knowledge that PINK1 is responsible for the phosphorylation of the subunit NDUFA10 of the major ETC complex and that PINK1-PD mutations have Complex I deficiencies, created a new link between sporadic and familiar forms of PD. Understanding deregulated mitochondrial functions through the role of PINK1 mutations in familial forms of PD, may elucidate whether these pathways are eventually also disrupted in sporadic cases. More recently, emerging data focus on PINK1 role in non-neuronal cells, giving rise to the concept that loss of neurons in PD may be due to a brain environmental issue and not neuronal per se. We consider that research, with for instance new culture models, is needed to elucidate the mechanisms underlying PD and to develop potential biomarkers for diagnostic purpose and neuroprotective strategies.

## Data Availability

Not applicable.
